# Emerging trends and focus on immune checkpoint inhibitors for non-small cell lung cancer treatment: visualization and bibliometric analysis

**DOI:** 10.3389/fphar.2023.1140771

**Published:** 2023-05-04

**Authors:** Yue Zhang, Lishan Lu, Rui Zheng

**Affiliations:** ^1^ Department of Laboratory Medicine, Shengjing Hospital of China Medical University, Shenyang, China; ^2^ Liaoning Clinical Research Center for Laboratory Medicine, Shenyang, China; ^3^ Department of Pulmonary and Critical Care Medicine, Shengjing Hospital of China Medical University, Shenyang, China

**Keywords:** non-small cell lung carcinoma, immune checkpoint inhibitors, bibliometric analysis, CiteSpace, VOSviewer

## Abstract

**Introduction:** Lung cancer is the leading cause of cancer-related deaths worldwide, and non-small cell lung carcinoma (NSCLC) accounts for approximately 80% of all cases. Immune checkpoint inhibitors (ICIs) are widely used to treat NSCLC owing to their remarkable efficacy. In this study, we analyzed the scientific collaboration network, defined the hotspots of research on the use of ICIs for NSCLC treatment, analyzed its evolution over the past few years, and forecasted the field’s future development using bibliometric analysis and a graphical study.

**Methods:** Research articles and reviews regarding ICIs for NSCLC were retrieved and obtained from the Web of Science Core Collection on 26 September 2022. CtieSpace and VOSviewer were thereafter used to conduct the bibliometric and knowledge-map analysis.

**Results:** We included 8,149 articles for this literature analysis. Our analysis showed that the USA had the highest number of publications and citations. We also noted that research trends in this field have changed drastically over the past 20 years, from the early development of ICIs, such as CTLA-4 inhibitors, to the development of recent ones, such as PD-1 and PD-L1 blockers. Further, the focus of research in this field has also gradually shifted from mechanisms to treatment effects and adverse events, suggesting that the field is maturing. Clinical applications are also being explored, including studies on how to enhance efficacy, reduce adverse effects, and expand to other specific cancer types.

**Conclusion:** To the best of our knowledge, this is the first study to construct a comprehensive knowledge map on ICIs for NSCLC. It can help researchers rapidly grasp the status and focus of current research in this area, offer direction, and serve as a reference for conducting similar studies.

## 1 Introduction

Lung cancer is the leading cause of cancer-related deaths globally (Bray et al., 2018), and small cell carcinoma (SCLC) accounts for approximately 20% of all cases, whereas non-small cell lung carcinoma (NSCLC), which includes the subtypes, adenocarcinoma (AD), squamous cell carcinoma, and large cell carcinoma, accounts for approximately 80% of all cases. The prognosis is poor for over 70% of patients with NSCLC, who are initially diagnosed when the disease is already at an advanced stage (Bodor et al., 2020). The 5-year survival rate of patients with advanced NSCLC is less than 3% (Ozkaya et al., 2012). In the past, chemotherapy regimens combined with radiotherapy were the recommended standard of care for patients with NSCLC who presented with advanced-stage disease (De Ruysscher et al., 2020). With a median progression-free survival (PFS) of only 4–6 months and overall survival (OS) of approximately 12–18 months, the treatment provided relatively modest responses (Assi et al., 2018; Suresh et al., 2018). Recent advancements in immunotherapy, which boosts antitumor activity using cytotoxic T-lymphocyte antigen 4 (CTLA-4), programmed cell death-1 (PD-1), and PD ligand 1 (PD-L1) inhibitors, have altered the therapeutic approach for a variety of cancers (Naidoo et al., 2020). Specifically, tumor immunotherapy, a cutting-edge and effective therapeutic approach for advanced NSCLC, has several potential applications (Lurienne et al., 2020; De Giglio et al., 2021). Immune checkpoint inhibitors (ICIs) are a class of immunotherapeutic agents capable of harnessing intrinsic immune response against tumor antigens by inhibiting T-cell activation by antigen-presenting cells. The first checkpoint to be identified was CTLA-4, and an ICI, ipilimumab, was developed to improve OS in patients with previously treated metastatic melanomas (Hodi et al., 2010; Larkin et al., 2015; Robert et al., 2015; Weber et al., 2017). Over the past few years, immunotherapeutic drugs that target immune checkpoint pathways have made significant strides in clinical trials and have quickly become the standard of care for advanced-stage NSCLC (Guibert and Mazières, 2015; Socinski et al., 2021; Reck and Hellmann, 2022).

Bibliometrics, which was first used in 1969, is a strategy for comprehensively reviewing a subject of study using mathematical and statistical techniques to examine literature statistically (Pritchard, 1969). This strategy is predominantly used in healthcare research to assess the influence or impact of research papers. Thus, the results of bibliometric studies, which analyze the influence or impact of a particular research paper on subsequent studies, are particularly useful for subjects that are progressively gaining interest. Traditional reviews usually reflect the latest developments in one aspect of a subject rather than the overall picture of the discipline. However, bibliometrics is a measurable informatics approach that addresses these limitations by analyzing the structure of knowledge related to a particular discipline to obtain quantifiable data. This multi-perspective, time-phased, and dynamic technique of visual analysis of literature can show the evolution of knowledge disciplines and automatically identify the research frontiers of the discipline through citation nodes and co-citation clustering, providing an important and viable systematic method for determining the importance of published literature by showing author networks and scholarly communication, connections between scholars, and developments in a field of knowledge.

Although bibliometric analysis has been used in several other domains, there is currently no bibliometric study on the use of ICIs for NSCLC treatment (Chen et al., 2020; Xin et al., 2021; Shen et al., 2022). To perform knowledge mapping and discover the hotspots or frontiers on ICIs for NSCLC treatment, we used CiteSpace, VOSviewer, and R tools in this study to create scientific knowledge maps and analyze publications from 2000 to 2022. The clinical trial characteristics were reviewed, and their development processes were summarized and visually represented in this study. We believe that this study can serve as a resource for future research.

## 2 Materials and methods

### 2.1 Data collection and search strategy

The most well-known and important database of scientific literature, the Web of Science Core Collection (WoSCC), was selected as the data source for this study. WoSCC, the most widely used database in prior bibliometric studies (Dai et al., 2022; Miao et al., 2022; Qin et al., 2022), is an ideal database containing information corresponding to over 10,000 high-quality journals. It was chosen for this bibliometric analysis because it offers a wealth of bibliometric indicators, such as publication and reference information. For this study, data were retrieved and downloaded from WoSCC on 26 September 2022. We set the search formula as follows: Immune checkpoint inhibitors search term: #1:TS=(ipilimumab) OR TI=(ipilimumab) OR AB=(ipilimumab); #2:TS=(pembrolizumab) OR TI=(pembrolizumab) OR AB=(pembrolizumab); #3:TS=(nivolumab) OR TI=(nivolumab) OR AB=(nivolumab); #4:TS=(immunotherapy) OR TI=(immunotherapy) OR AB=(immunotherapy) OR TS=(immune checkpoint blockade) OR TI=(immune checkpoint blockade) OR AB=(immune checkpoint blockade) OR TS=(immune checkpoint inhibitor) OR TI=(immune checkpoint inhibitor) OR AB=(immune checkpoint inhibitor); #5:TS=(PD-1) OR TI=(PD-1) OR AB=(PD-1) OR TS=(PD-L1) OR TI=(PD-L1) OR AB=(PD-L1) OR TS=(CTLA-4) OR TI=(CTLA-4) OR AB=(CTLA-4); #6; TS=(yervoy) OR TI=(yervoy) OR AB=(yervoy) OR TS=(keytruda) OR TI=(keytruda) OR AB=(keytruda) OR TS=(opdivo) TI=(opdivo) OR AB=(opdivo); #7:#1 OR #2 OR #3 OR #4 OR #5 OR #6; non-small cell lung cancer search term: #8: TS=(non-small cell lung cancer) OR TI=(non-small cell lung cancer) OR AB=(non-small cell lung cancer); #9:TS=(non-small cell lung carcinoma) OR TI=(non-small cell lung carcinoma) OR AB=(non-small cell lung carcinoma); #10:TS=(NSCLC) OR TI=(NSCLC) OR AB=(NSCLC); #11:#8 OR #9 OR #10. Final Data Sources: #7 and #11.

### 2.2 Inclusion and exclusion criteria

**FIGURE 1:**
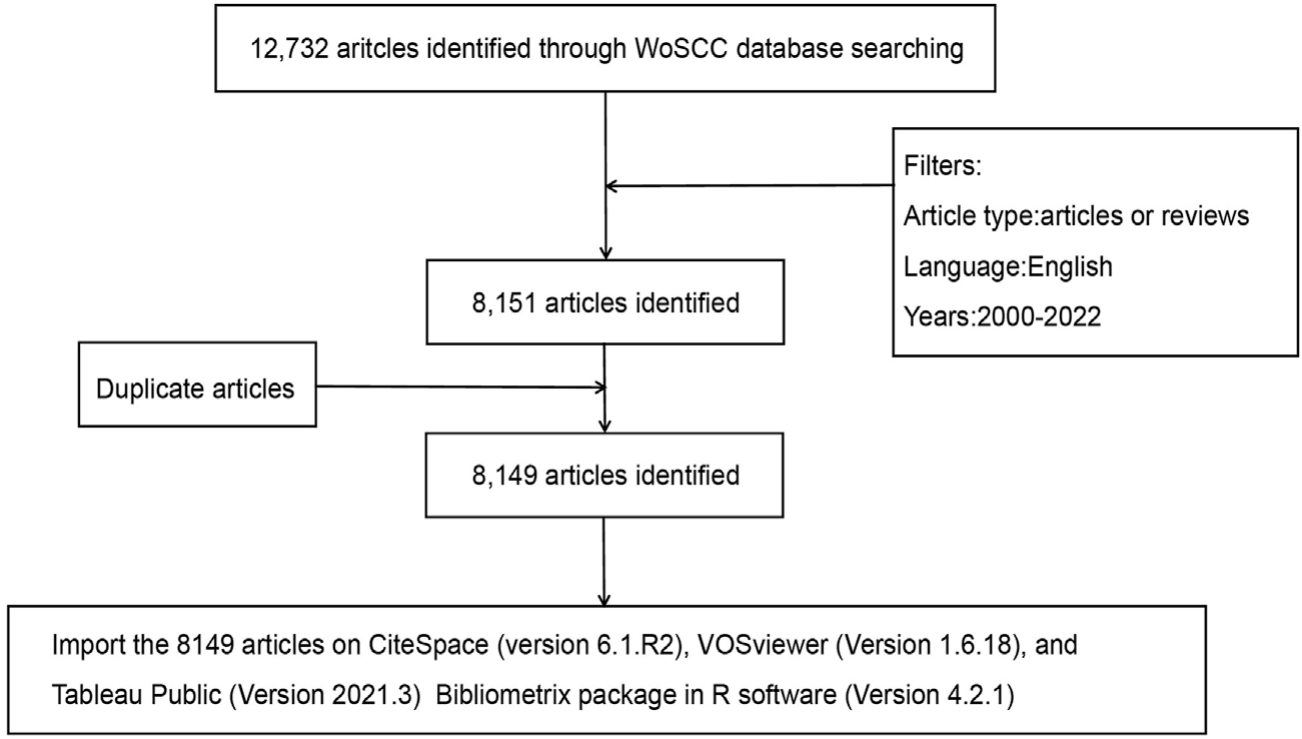
Flowchart of the literature search and screening procedures used in the study.

The study period was from 1 January 2000, to 26 September 2022, with English as the only permitted language. The only available documents were research and review articles. Figure 1 displays a flowchart of the study’s procedures.

### 2.3 Data analysis and visualization

CiteSpace (version 6.1. R2), VOSviewer (version 1.6.18), Tableau Public (version 2021.3), and R (version 4.2.1) software were used for data analysis and visualization. CiteSpace, created in 2004 by Chaomei Chen at Drexel University, is typically used to analyze, spot, and display trends and patterns in scientific publications (Chen, 2004). Thus, researchers can use it to predict the research and development trend in their area of interest and facilitate an intuitive understanding of research hotspots and the evolution process. In this study, it was primarily used to visualize articles that had the largest number of citations. Further, VOSviewer, which is more focused on the visualization of scientific information, is a Java-based bibliometric mapping program that has a powerful capability to handle large maps and display large bibliometric maps in an easily interpretable manner (van Eck and Waltman, 2010). In this study, co-authorship analysis in terms of country, author, and institution; co-citation analysis of the journal and author; and keyword co-occurrence analysis were performed using VOSviewer. The spatial distribution of the global publications was realized using the Tableau Public software. Further, the Bibliometrix package in R software offered the possibility to visualize the keywords with the largest number of citations. Together, these analytical tools offered unbiased and varied perspectives on the advancement of the use of ICIs for NSCLC treatment.

## 3 Results

### 3.1 Obtaining relevant literature

A total of 12,732 articles were first identified in the WoSCC database. According to the exclusion criteria, 3,803 meeting abstracts, 401 editorial materials, 151 letters, 67 revisions, 53 online publications, and 6 other categories of literature were excluded (a total of 4,481 articles). Further, a total of 100 non-English articles (49 in German, 41 in French, 4 in Spanish, 3 in Polish, 1 in Hungarian, 1 in Japanese, and 1 in Portuguese) and 2 duplicate articles were also excluded (Figure 1).

### 3.2 Analysis of annual publications and citation trends

**FIGURE 2:**
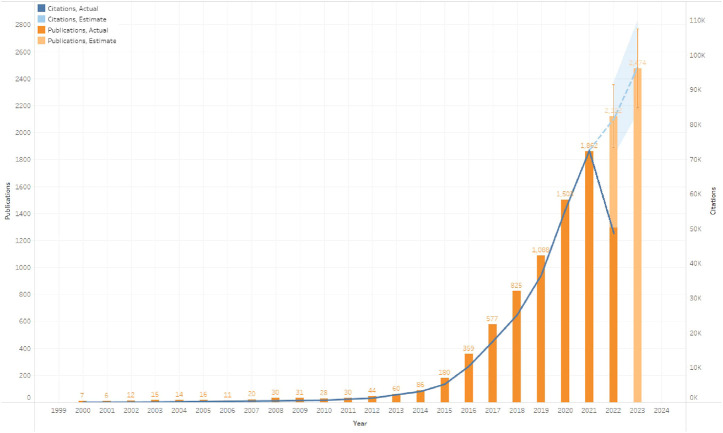
Global trend of publications and total citations on the use of immune checkpoint inhibitors (ICIs) for non-small cell lung cancer (NSCLC) treatment from 2000 to 2022.

From 2000 to 2022, 8,149 research and review articles were published on the use of ICIs for NSCLC treatment. We noted that between 2000 and 2014, only 410 were published articles, and these showed no evident research trends, i.e., the number of publications remained relatively constant. As more scholars focused on ICIs for NSCLC treatment, the annual growth rate of papers varied. After 2015, the annual production of relevant papers exhibited a sharp increase, peaking in 2021. There were approximately 60 times more publications in 2021 (1,862) than in 2011 (30). We also constructed models based on exponential smoothing to estimate the number of papers that will be published and cited in 2022 and 2023, forecasted at approximately 2,122 and 2,474 publications, respectively (Figure 2) (JO, 1971).

### 3.3 Analysis of countries/regions

**FIGURE 3:**
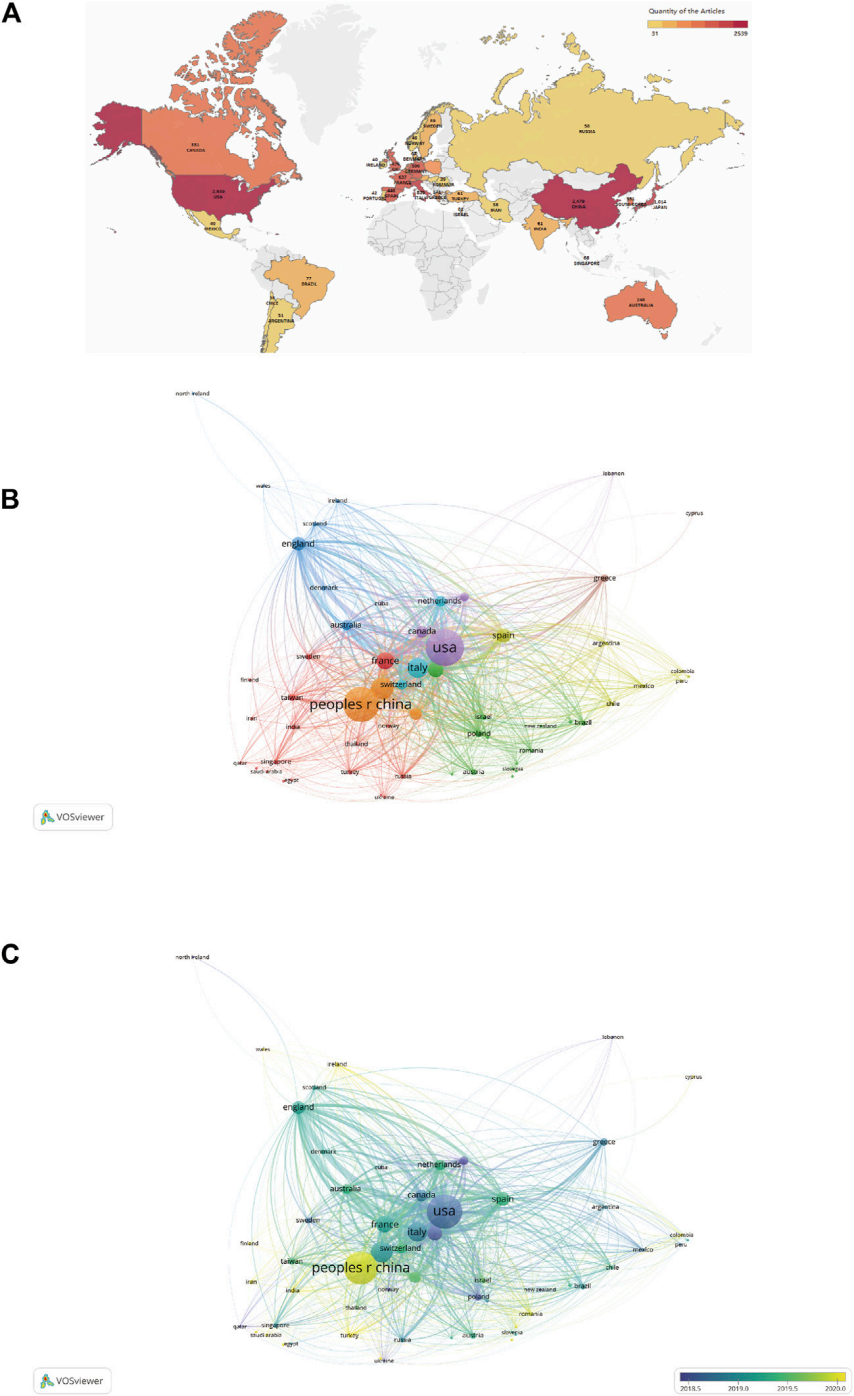
**(A)** Geographic distribution map based on publications from different countries/regions (n ≥ 30). **(B)** Countries/regions citation network visualization map. The number of publications is represented by the size of each circle/node. Further, the strength of the connections between the circles/nodes is represented by the thickness of the connecting lines, and each color represents a cluster, i.e., a group of objects with related characteristics within a network. Each circle or node represents a separate nation or region. **(C)** Countries/regions citation overlay visualization map. The institutions that earlier commenced research in this field of research are depicted using purple nodes, whereas the yellow nodes represent institutions that commenced research in this field later.

Thirty-five countries with more than 30 publications in the field of ICIs for NSCLC treatment were identified (Figure 3A). The top ten productive nations/regions were ranked, as shown in Table 1. Notably, the top five nations were the USA (2,539/31.2%), China (mainland) (2,341/28.7%), Japan (1,041/12.8%), Italy (839/10.3%), and France (637/7.8%), with the USA taking the top spot. Although the number of publications in China was similar to that in the USA, the total number of citations (36,035) was much lower than that for the USA (168,045). VOSviewer was used to build a co-authorship network map to study the cooperation among countries/regions. The map of global collaboration shows how closely nations were working together. The two nations with the most international partnerships were the USA and China (Figure 3B). Further, the VOSviewer-based overlay visualization map of organizational cooperation was used to analyze the publication time. Notably, China started publishing more recently than most other productive nations (Figure 3C).

**TABLE 1:** Top 10 countries/regions contributing to publications of ICIs for NSCLC.

Country/region	Publication	Times Cited (total)	Times Cited (without self-citations)	Citations per publication	H-index
United States of America	2539	185084	168045	72.9	170
CHINA(mainland)	2341	41927	36035	17.91	87
JAPAN	1014	42718	39433	42.13	80
ITALY	839	48303	45220	57.57	77
FRANCE	637	50286	48134	78.94	86
GERMANY	506	56102	54521	110.87	76
SPAIN	446	59570	57475	133.57	80
ENGLAND	407	26695	26025	65.59	70
CANADA	331	33083	32226	99.95	65
SOUTH KOREA	331	27067	26384	81.77	58

### 3.4 Analysis of productive organizations

**FIGURE 4:**
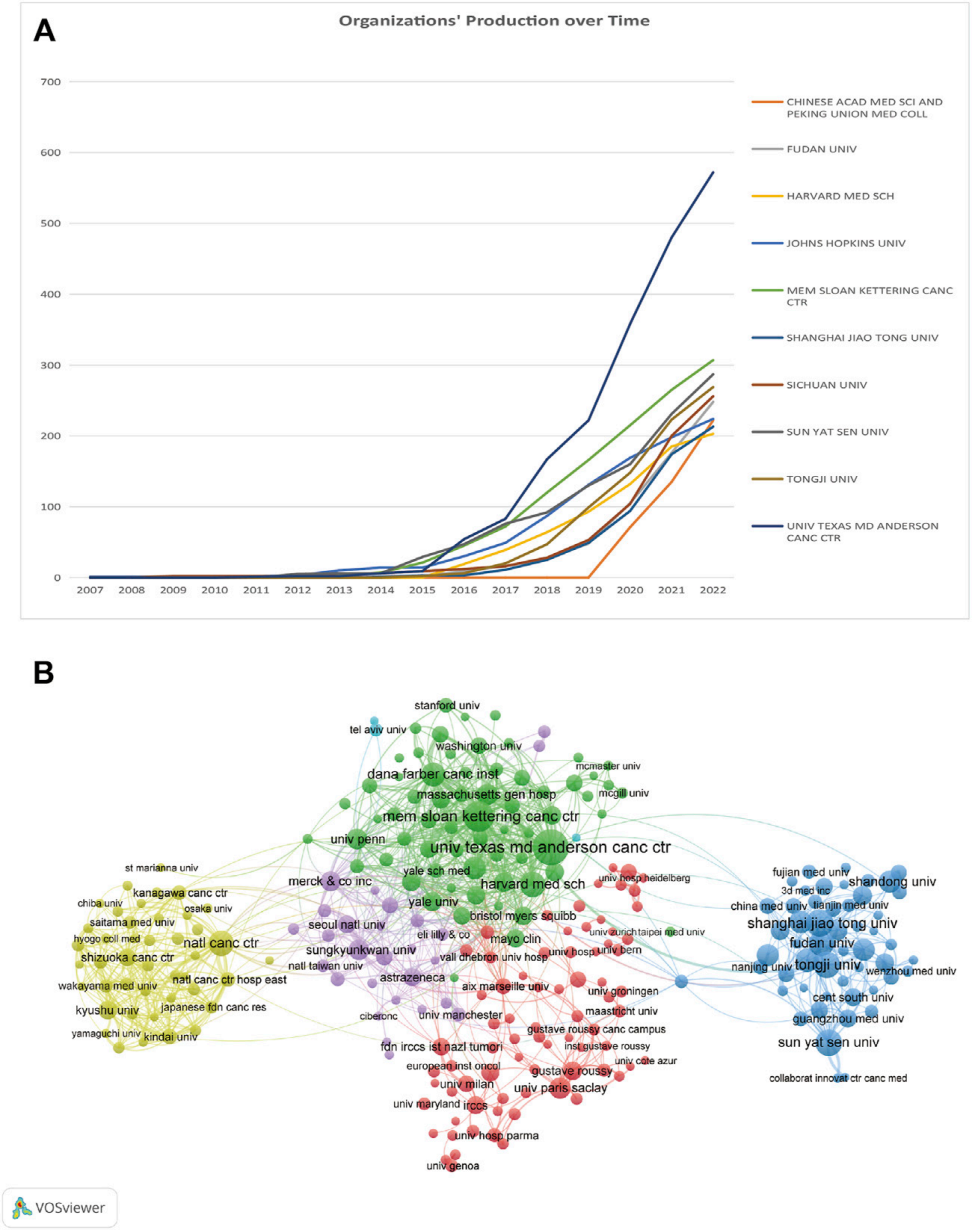
**(A)** Chronological changes in the number of publications from the top 10 organizations. **(B)** Network visualization maps of organizations.

In total, 8,149 documents were released by 8,353 institutions. Further, 265 organizations that qualified for inclusion (publications >20) were included in the visualization analysis. Table 2 shows the institutions with the highest number of publications and citations. The top three productive organizations were the University of Texas MD Anderson Cancer Center (572/7.0%), the Memorial Sloan-Kettering Cancer Center (307/3.8%), and SUN YAT-SEN University(293/3.5%). Four of the top 10 institutions were US-based, while the remaining six were in China. Regarding organizations with the most citations, the top three were the Memorial Sloan-Kettering Cancer Center (37,968 citations), Dana-Farber Cancer Institute (28,147 citations), and Yale University (25,653 citations). Further, eight of the top 10 institutions were US-based. The cumulative annual publications of the top 10 institutions are shown in Figure 4A. We also examined the organizations’ co-authorship (Figure 4B). VOSviewer was used to build a co-authorship network map of countries to study cooperation among organizations. All 265 top publishing institutions could be divided into six clusters, each roughly denoting one country. The National Cancer Institute, Memorial Sloan Kettering Cancer Center, and MD Anderson Cancer Center are a few American institutions represented by the green clusters. With limited connections to other clusters, the deep blue cluster represented Chinese universities and institutions, including SUN YAT-SEN University and Tongji University. Universities and other institutions in Japan are represented using the yellow cluster.

**TABLE 2:** Top 10 productive organizations and the top 10 organizations with the most citations in the field of ICIs for NSCLC.

Rank	Organization	Publication	Country	Rank	Organization	Citations	Country
1	UNIV TEXAS MD ANDERSON CANC CTR	572	United States of America	1	MEM SLOAN KETTERING CANC CTR	37968	United States of America
2	MEM SLOAN KETTERING CANC CTR	307	United States of America	2	DANA-FARBER CANCER INSTITUTE	28147	United States of America
3	SUN YAT SEN UNIV	293	China	3	YALE UNIV	25653	United States of America
4	TONGJI UNIV	269	China	4	JOHNS HOPKINS UNIV	21879	United States of America
5	SICHUAN UNIV	258	China	5	UNIV TEXAS MD ANDERSON CANC CTR	18266	United States of America
6	FUDAN UNIV	248	China	6	WEILL CORNELL MED COLL	17891	United States of America
7	JOHNS HOPKINS UNIV	224	United States of America	7	UNIV CALIF LOS ANGELES	17165	United States of America
8	CHINESE ACAD MED SCI AND PEKING UNION MED COLL	223	China	8	SUNGKYUNKWAN UNIV	16030	Korea
9	SHANGHAI JIAO TONG UNIV	212	China	9	COLUMBIA UNIV	14870	United States of America
10	HARVARD MED SCH	203	United States of America	10	UNIV SYDNEY	14002	Australia

### 3.5 Analysis of authors and Co-cited authors

**FIGURE 5:**
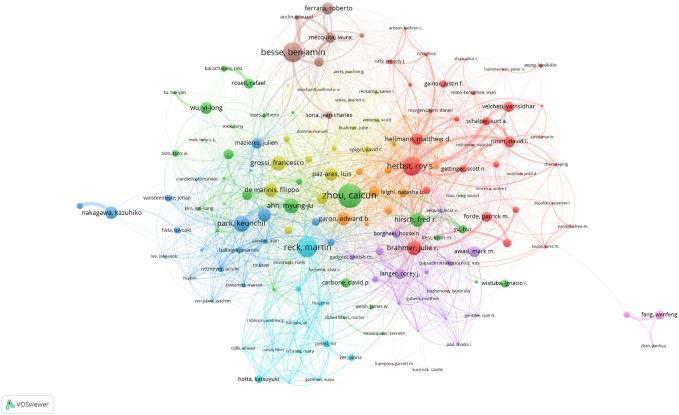
Network visualization map of author co-authorship analysis.

More than 30,000 researchers have been involved in the study of ICIs for NSCLC treatment. Among these, the top three authors with the most publications were Caicun Zhou (68), Martin Reck (57), and Benjamin Besse (55) (Table 3). Among the top 10 most-cited authors (Table 3), Julie R Brahmer, Martin Reck, and Gregory M Lubiniecki (total citations: 13,819, 13,669, and 10,803) ranked first, second, and third, respectively. Additionally, as shown in Figure 5, each author (n > 5, total citations >100) is represented by a single node, while authors with large publishing volumes, such as Caicun Zhou, Martin Reck, and Benjamin Besse, created several autonomous core author groups. These groups exhibited close collaboration and few ties with other author groups.

**TABLE 3:** The 10 most productive authors and the top 10 authors with most citations in the field of ICIs for NSCLC.

Rank	Author	Articles	Rank	Author	Total citations
1	Caicun Zhou	68	1	Julie R Brahmer	13819
2	Martin Reck	57	2	Martin Reck	13669
3	Benjamin Besse	55	3	Gregory M Lubiniecki	10803
4	Roy S Herbst	52	4	Edward B Garon	10691
5	Yuichiro Ohe	46	5	Roy S Herbst	9462
6	Keunchil Park	45	6	Paul Baas	9090
7	Myung-Ju Ahn	42	7	Matthew D Hellmann	8669
8	Francesco Grossi	42	8	Naiyer A Rizvi	8492
9	Jinming Yu	41	9	Rina Hui	7234
10	Julie R Brahmer	40	10	Delvys Rodríguez-Abreu	7088

### 3.6 Analysis of influential journals and Co-cited journals

**FIGURE 6:**
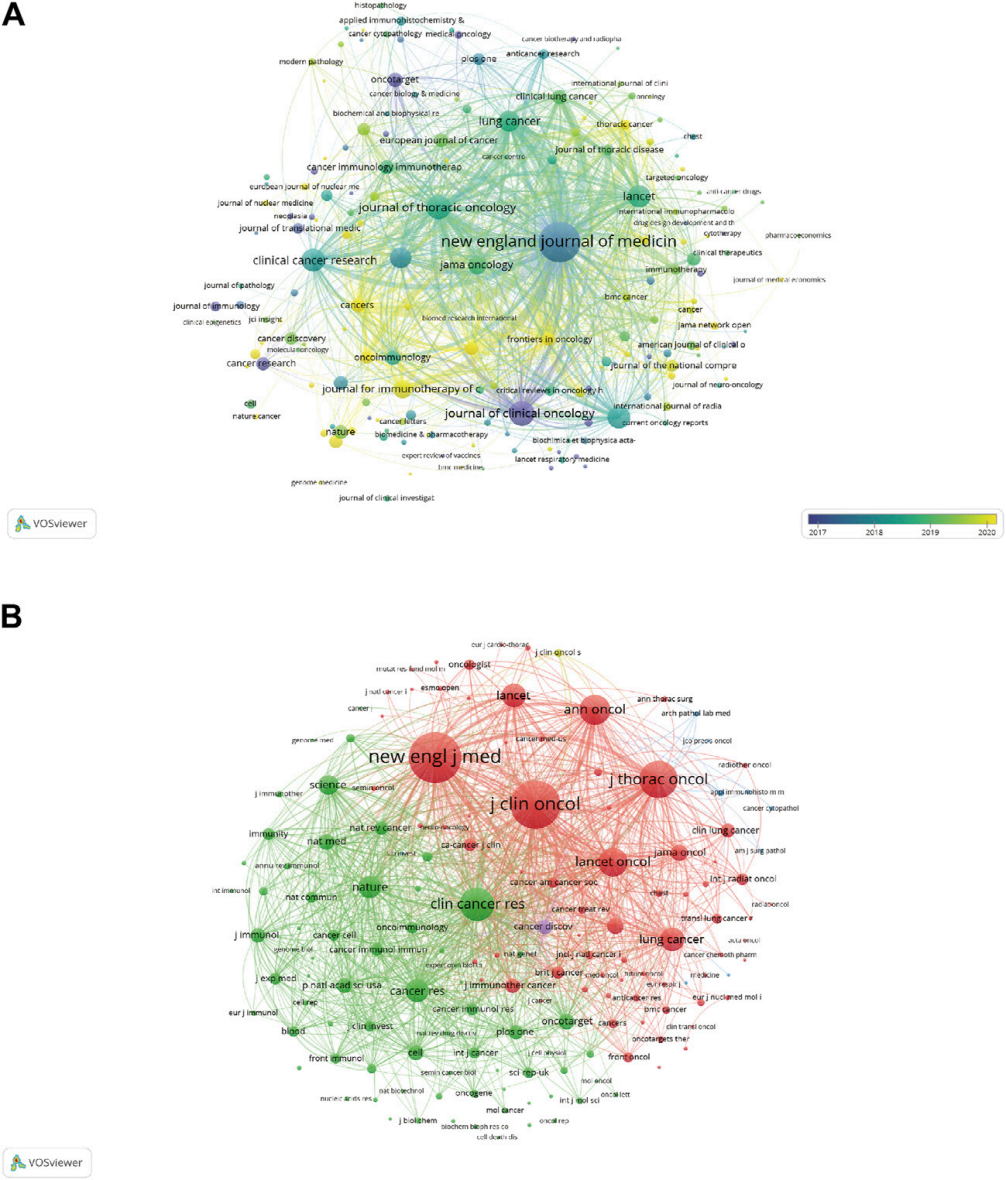
**(A)** Overlay visualization maps of citing journals. **(B)** Network visualization maps of co-cited journals.

A total of 890 journals were included in this study, with Frontiers in Oncology (N = 336, impact factor (IF) = 5.74, Q2), Lung Cancer (N = 327, IF = 6.08, Q1), and Cancers (N = 276, IF = 6.57, Q1) identified as the top three journals with the highest number of publications (Table 4). Journal of Thoracic Oncology had the highest IF among the 10 most productive journals (N = 205, IF = 20.12, Q1). Further, all the top 10 co-cited publications, shown in Table 4, received more than 6,500 citations, with New England Journal of Medicine *,* Journal of Clinical Oncology *,* Journal of Thoracic Oncology *,* Clinical Cancer Research *,* and Annals of Oncology identified as the top five most co-cited journals (total citations: 27,740, 25,094, 16,166, 13,823, and 11,487, respectively). The network visualization maps of the citing and co-cited journals were generated using VOSviewer. As shown in Figures 6A, B, many journals co-occurred in both maps and had live citation links.

**TABLE 4:** Top 10 productive journals and co-cited journals in the field of ICIs for NSCLC.

Rank	Journals	Counts	IF (2021)	JCR (2021)	Co-cited journals	Total citations	IF (2021)	JCR (2021)
1	Frontiers in Oncology	336	5.74	Q2	New England Journal of Medicine	27740	176.07	Q1
2	Lung Cancer	327	6.08	Q1	Journal of Clinical Oncology	25094	50.72	Q1
3	Cancers	276	6.57	Q1	Journal of Thoracic Oncology	16166	20.12	Q1
4	Translational Lung Cancer Research	201	4.73	Q2	Clinical Cancer Research	13823	13.8	Q1
5	Journal of Thoracic Oncology	205	20.12	Q1	Annals of Oncology	11487	51.77	Q1
6	Clinical Lung Cancer	202	4.84	Q2	Lancet Oncology	10697	54.43	Q1
7	Journal for Immunotherapy of Cancer	202	12.47	Q1	Lancet	7829	202.73	Q1
8	Thoracic Cance	191	3.22	Q3	Lung Cancer	7827	6.08	Q1
9	Frontiers in Immunology	166	8.79	Q1	Cancer Research	7337	13.31	Q1
10	Clinical Cancer Research	135	13.8	Q1	Nature	6976	69.5	Q1

### 3.7 Analysis of references and Co-cited references

**FIGURE 7:**
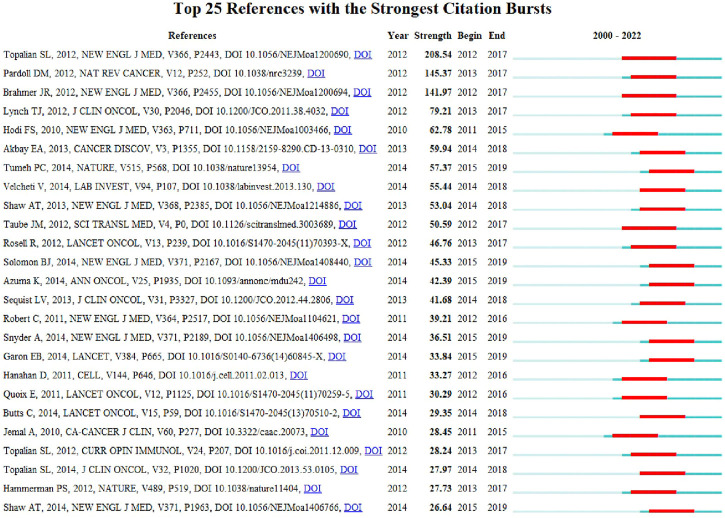
Top 25 references with strongest publication bursts.

In this study, reference analysis was performed to understand advances in research on the use of ICIs in NSCLC treatment. Table 5 outlines the top 10 cited and/or co-cited references. The titles of the top three most frequently cited articles were “Safety, Activity, and Immune Correlates of Anti-PD-1 Antibody in Cancer,” authored by Suzanne L. Topalian (Topalian et al., 2012) (total citations: 8,542; Publication Year: 2012); “Nivolumab *versus* Docetaxel in Advanced Non-squamous Non-small Cell Lung Cancer,” authored by Hossein Borghaei (Borghaei et al., 2015) (total citations: 6,110; Publication Year: 2015); and “Pembrolizumab *versus* Chemotherapy for PD-L1-Positive Non-Small-Cell Lung Cancer,” authored by Martin Reck (Reck et al., 2016) (total citations: 5,560; Publication Year: 2016). Seven of the top ten most-cited articles were published in the New England Journal of Medicine. As a result, articles with the highest number of citations were examined, and CiteSpace was used to display the network of articles’ co-citations. In addition, articles on ICIs for NSCLC treatment with the strongest citation bursts were identified using CiteSpace. The top 25 images are shown in Figure 7. Further, the titles of the top three articles with the most frequent citation bursts were “Safety, Activity, and Immune Correlates of Anti-PD-1 Antibody in Cancer” (Topalian et al., 2012) (Strength: 208.54; Publication Year: 2012), “The Blockade of Immune Checkpoints in Cancer Immunotherapy” (Pardoll, 2012) (Strength: 145.37; Publication Year: 2012), and “Safety and Activity of Anti-PD-L1 Antibody in Patients with Advanced Cancer” (Brahmer et al., 2012) (Strength: 141.97; Publication Year: 2012). We also noted that the titles of the top three articles with the most frequent citation bursts were “Nivolumab *versus* Docetaxel in Advanced Non-squamous Non-Small-Cell Lung Cancer” (Borghaei et al., 2015) (Co-citations: 2,554; Publication Year: 2015), “Pembrolizumab *versus* Chemotherapy for PD-L1-Positive Non-Small-Cell Lung Cancer” (Reck et al., 2016) (Co-citations: 2,322; Publication Year: 2016), and “Nivolumab *versus* Docetaxel in Advanced Squamous-Cell Non-Small-Cell Lung Cancer” (Brahmer et al., 2015) (Co-citations: 1,896; Publication Year: 2015) (Table 6).

**TABLE 5:** Top 10 highly cited articles in the field of ICIs for NSCLC.

Rank	Paper title	First author	Year	Journal	TC	TC per year	DOI
1	Safety, Activity, and Immune Correlates of Anti–PD-1 Antibody in Cancer	Suzanne L. Topalian	2012	NEW ENGL J MED	8541	776.45	10.1056/NEJMoa1200690
2	Nivolumab *versus* Docetaxel in Advanced Non-squamous Non-Small-Cell Lung Cancer	Hossein Borghaei	2015	NEW ENGL J MED	6110	763.75	10.1056/NEJMoa1507643
3	Pembrolizumab *versus* Chemotherapy for PD-L1–Positive Non-Small-Cell Lung Cancer	Martin Reck	2016	NEW ENGL J MED	5560	794.29	10.1056/NEJMoa1606774
4	Safety and Activity of Anti–PD-L1 Antibody in Patients with Advanced Cancer	Julie R. Brahmer	2012	NEW ENGL J MED	5420	492.73	10.1056/NEJMoa1200694
5	Mutational landscape determines sensitivity to PD-1 blockade in non-small cell lung cancer	Naiyer A. Rizvi	2015	SCIENCE	5118	639.75	10.1126/science.aaa1348
6	Nivolumab *versus* Docetaxel in Advanced Squamous-Cell Non-Small-Cell Lung Cancer	Julie Brahmer	2015	NEW ENGL J MED	4723	590.38	10.1056/NEJMoa1504627
7	Pembrolizumab for the Treatment of Non-Small-Cell Lung Cancer	Edward B. Garon	2015	NEW ENGL J MED	3993	499.13	10.1056/NEJMoa1501824
8	Pembrolizumab *versus* docetaxel for previously treated, PD-L1-positive, advanced non-small-cell lung cancer (KEYNOTE-010): a randomised controlled trial	Roy S Herbst	2016	LANCET	3330	475.71	10.1016/S0140-6736 (15)01281–7
9	Pembrolizumab plus Chemotherapy in Metastatic Non-Small-Cell Lung Cancer	Leena Gandhi	2018	NEW ENGL J MED	3049	609.80	10.1056/NEJMoa1801005
10	Atezolizumab *versus* docetaxel in patients with previously treated non-small-cell lung cancer (OAK): a phase 3, open-label, multicentre randomised controlled trial	Achim Rittmeyer	2017	LANCET	2790	465.00	10.1016/S0140-6736 (16)32517-X

TC, total citations.

**TABLE 6:** The top 10 co-cited references in the field of ICIs for NSCLC.

Rank	Paper title	First author	Year	Journal	Co-citation	DOI
1	Nivolumab *versus* Docetaxel in Advanced Non-squamous Non-Small-Cell Lung Cancer	Hossein Borghaei	2015	NEW ENGL J MED	2554	10.1056/NEJMoa1507643
2	Pembrolizumab *versus* Chemotherapy for PD-L1–Positive Non-Small-Cell Lung Cancer	Martin Reck	2016	NEW ENGL J MED	2322	10.1056/NEJMoa1606774
3	Nivolumab *versus* Docetaxel in Advanced Squamous-Cell Non-Small-Cell Lung Cancer	Julie Brahmer	2015	NEW ENGL J MED	1896	10.1056/NEJMoa1504627
4	Pembrolizumab *versus* docetaxel for previously treated, PD-L1-positive, advanced non-small-cell lung cancer (KEYNOTE-010): a randomised controlled trial	Roy S Herbst	2016	LANCET	1637	10.1016/S0140-6736 (15)01281–7
5	Atezolizumab *versus* docetaxel in patients with previously treated non-small-cell lung cancer (OAK): a phase 3, open-label, multicentre randomised controlled trial	Achim Rittmeyer	2017	LANCET	1511	10.1016/S0140-6736 (16)32517-X
6	Pembrolizumab plus Chemotherapy in Metastatic Non-Small-Cell Lung Cancer	Leena Gandhi	2018	NEW ENGL J MED	1403	10.1056/NEJMoa1801005
7	Pembrolizumab for the Treatment of Non-Small-Cell Lung Cancer	Edward B. Garon	2015	NEW ENGL J MED	1275	10.1056/NEJMoa1501824
8	Mutational landscape determines sensitivity to PD-1 blockade in non-small cell lung cancer	Naiyer A. Rizvi	2015	SCIENCE	1034	10.1126/science.aaa1348
9	Pembrolizumab plus Chemotherapy for Squamous Non-Small-Cell Lung Cancer	Luis Paz-Ares	2018	NEW ENGL J MED	874	10.1056/NEJMoa1810865
10	Safety, Activity, and Immune Correlates of Anti–PD-1 Antibody in Cancer	Suzanne L. Topalian	2012	NEW ENGL J MED	874	10.1056/NEJMoa1200690

### 3.8 Emerging Trends and Research Focus Based on Keywords Analysis

**FIGURE 8:**
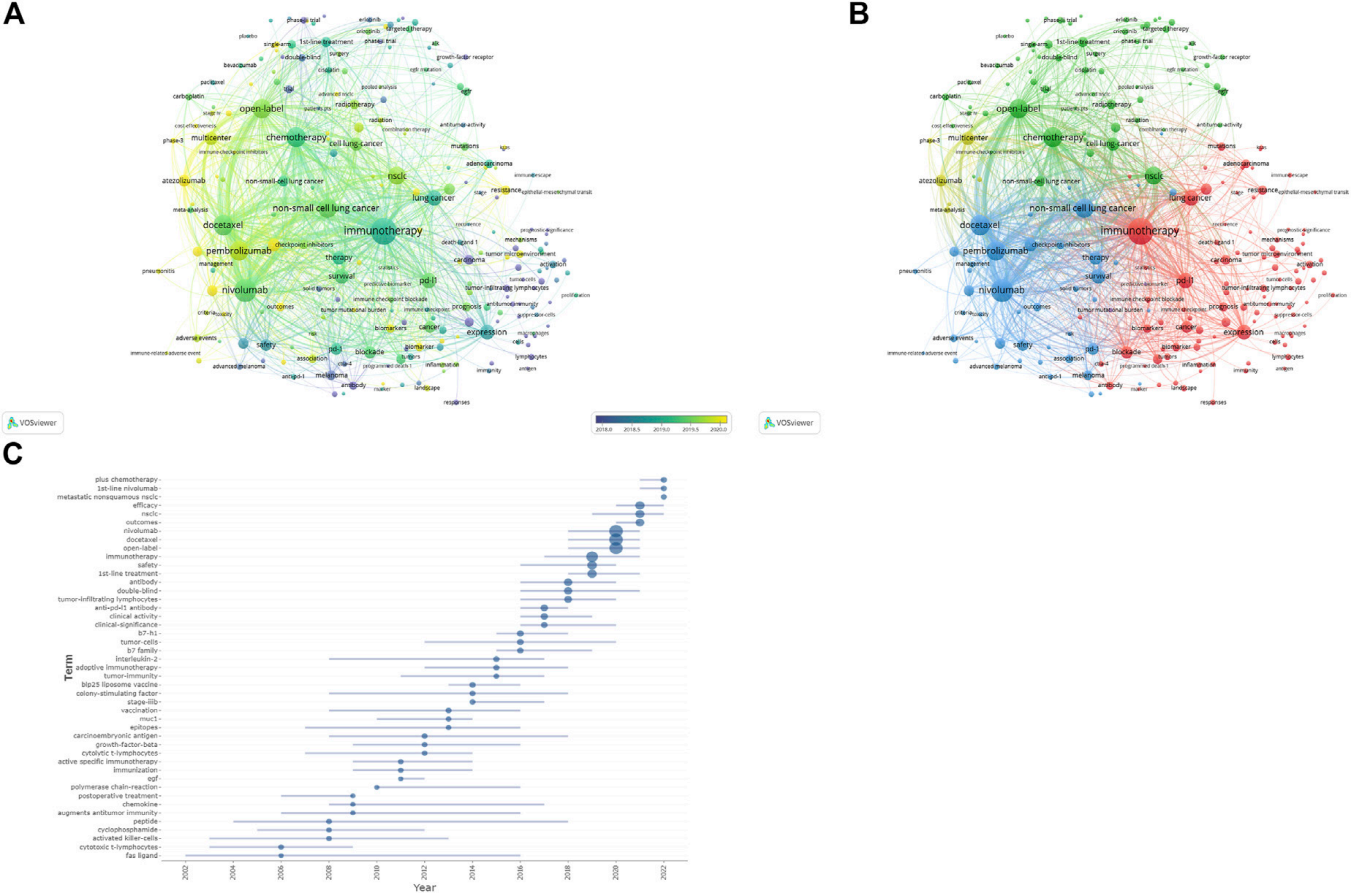
**(A)** Overlay visualization maps of keywords. **(B)** Network visualization maps of keywords. **(C)** Trend map of top 45 keywords.

**FIGURE 9:**
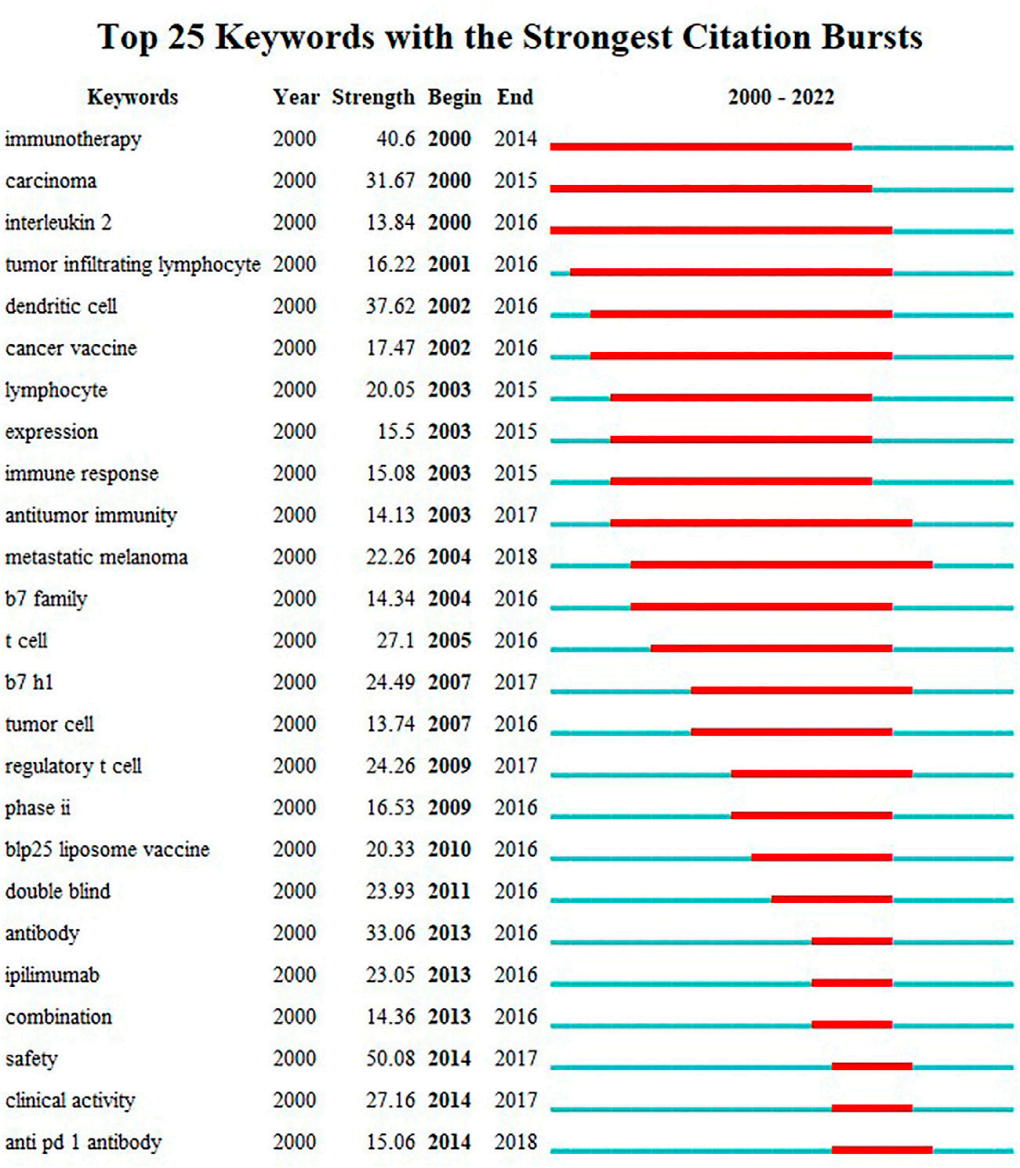
Top 25 keywords with the strongest citation bursts.

The visualization map of keywords was generated using VOSviewer software after combining the synonyms and eliminating irrelevant terms. Thus, a total of 13,814 keywords were included, and 220 terms with at least 50 occurrences were identified and grouped based on publication date (2018–2020) (Figure 8A) and into four clusters (Figure 8B). According to the sequence in which these technologies advance, hot research regions changed with time, transitioning from “antibody” to “atezolizumab,” “pneumonitis,” and so on, as seen by the overlay map of keywords classified based on publication date. Further, the keywords were separated into four clusters for the network map. One of the clusters shown in red included “lung cancer,” “PD-L1,” “cancer,” and “immunotherapy.” The other cluster, shown in green, generally contained terms associated with clinical oncology issues, such as “radiotherapy,” “chemotherapy,” “double-blind,” and “open-label.” The blue cluster included the names of antibodies approved by the US Food and Drug Administration (FDA), such as “nivolumab,” “pembrolizumab,” “atezolizumab,” and “ipilimumab,” and the yellow cluster included “multicenter” and “phase 3". The top 45 terms in the keyword trend map are shown in Figure 8C, from which it is evident that the keywords have undergone a significant shift recently. In recent years (2021–2022), researchers have been interested in “plus chemotherapy,” “1^st^ line nivolumab,” and other aspects. Additionally, Table 7 outlines the top 20 co-occurrence author keywords frequently appearing in this study, including nivolumab and docetaxel. The burst for keywords revealed that “immunotherapy” is still the research front in the field of ICIs for NSCLC (Figure 9).

**TABLE 7:** Top 20 co-occurrence keywords involved in the field of ICIs for NSCLC.

Rank	Keywords	Occurrences	Rank	Keywords	Occurrences
1	nivolumab	1810	11	cell lung-cancer	744
2	docetaxel	1783	12	blockade	664
3	open-label	1647	13	safety	515
4	chemotherapy	1419	14	cancer	496
5	pembrolizumab	1241	15	pd-l1 expression	481
6	immunotherapy	1108	16	1st-line treatment	442
7	expression	963	17	ipilimumab	437
8	survival	825	18	atezolizumab	427
9	multicenter	755	19	efficacy	421
10	therapy	748	20	pd-1 blockade	389

## 4 Discussion

To the best of our knowledge, this is the first bibliometric analysis comparing the outcomes of ICIs in NSCLC treatment. We performed a bibliometric analysis involving 8,149 articles from the WoSCC database corresponding to the 2000–2022 period. We further summarized the research themes, trends, and sources of ICIs for NSCLC treatment and the global impact of research in this field.

One of the most prevalent cancers in both men and women is NSCLC, and treating it is still challenging on a global scale. Based on our results, the number of papers related to ICIs for NSCLC has been steadily increasing. This indicates that this subject has recently received considerable attention. Additionally, a notable trend shift occurred in 2015. This may be related to the FDA approval of the PD-1 checkpoint drugs (nivolumab and pembrolizumab) for the immunotherapy of NSCLC and melanoma at the end of 2014 (Borghaei et al., 2015; Herbst et al., 2016). This marked the beginning of a new era of ICI use for NSCLC. Our chronological distribution results indicated that the research field on the use of ICIs for NSCLC has steadily been a research hot zone and is currently undergoing a significant development stage. During this period, the theory advanced quickly, and the volume of papers also grew rapidly. Moreover, the growth curve was abrupt, and its gradient did not lessen in 2022. Therefore, it can be expected that in the upcoming years, this field would continue to advance.

The USA, China, Japan, Italy, and France published the most articles (Table 1), and the highest level of international cooperation corresponded to the USA (Figure 3). This may be due to the rapid economic development in these countries and increased investment in related fields, resulting in more publications. Additionally, cooperation with developed countries can help increase scientific productivity given that these developed countries have sophisticated medical standards and a wealth of resources for scientific research; therefore, to encourage the advancement of global ICI research, we recommend that developed countries strengthen their partnerships and connections with more nations and regions, particularly with those that are still relatively underdeveloped. More than a quarter of all publications came from China, the only developing nation listed among the top ten most productive nations or regions. Even though China is the second-largest publishing nation, none of the top ten journals were Chinese, suggesting that China should improve the quality of its periodic productions in this area. Only less than half of all the countries published more than 30 publications. Therefore, to ensure improvement in this area, we need to strengthen international cooperation while improving the quality of publications.

Institutional cooperation is an aggregative trend from the perspective of cooperative relationships. Most cooperative Chinese institutions are located in China. American institutions have collaborated more with other countries. More multi-institutional collaborations, such as academic exchanges and integration, are required to develop the field. This will help improve the overall level of institutions, and the leading institutions in the field will exchange advanced technologies and ideas with relatively less apt institutions.

Among the co-cited journals, the *New England Journal of Medicine* may be the most important journal on the use of ICIs for NSCLC treatment, according to the total number of citations and its IF. This is because clinical trials of ICIs for NSCLC treatment have been published in these journals, attracting a great deal of attention. Simultaneously, our findings provide a reference for researchers in this field to choose journals for submission and reading articles.

Regarding authorship, Martin Reck ranked second in the number of publications and the total number of citations. He also published the article with the third highest number of citations titled “Pembrolizumab *versus* Chemotherapy for PD-L1-Positive Non-Small-Cell Lung Cancer,” indicating his important role in this field. Martin Reck is a chief physician at the Lung Clinic Grosshansdorf, Germany, and principal investigator in KEYNOTE-024 (NCT02142738) (Reck et al., 2016; Brahmer et al., 2017; Reck et al., 2021), a phase 3 study for PD-L1-positive NSCLC, which compared pembrolizumab with platinum-based doublet chemotherapy. In patients with EGFR/ALK-negative advanced NSCLC with ≥50% PD-L1 tumor cell positivity, pablizumab significantly improved objective remission rates (44.8% vs. 27.8%), prolonged PFS (10.3 months vs. 6.0 months; HR = 0.50, 95% CI: 0.37–0.68) and OS (26.3 months vs. 13.4 months; HR = 0.62, 95% CI: 0.48–0.81), and exhibited a lower rate of grade 3 or higher treatment-related adverse events (26.6% vs. 53.3%) compared to their counterparts who received chemotherapy.

The study by Topalian et al. demonstrated that individuals with specific advanced malignancies, including NSCLC, experience long-lasting tumor reduction and disease stabilization when their PD-L1 was blocked by an antibody (Topalian et al., 2012). These results support the importance of targeting the PD-1–PD-L1 pathway as a therapeutic target for some patients with cancer. The second highly-cited article, authored by Hossein (Borghaei et al., 2015) and published in the *New England Journal of Medicine* (cited 6,110 times), demonstrated that nivolumab outperformed docetaxel in terms of OS, with patients with advanced non-squamous NSCLC for whom platinum-based chemotherapy had failed benefiting from the increased efficacy of this drug owing to PD-L1 expression. Compared with docetaxel, nivolumab has a better safety profile. The third highly-cited article, authored by Martin Reck (Reck et al., 2016) and also published in the *New England Journal of Medicine,* noted that in patients with advanced NSCLC exhibiting PD-L1 expression on at least 50% of tumor cells, pembrolizumab was associated with considerably longer PFS and OS and with fewer adverse effects than for the platinum-based treatment.

To analyze research trends, we also performed keyword and burst term analyses. Thus, we observed a significant change in focus over time. Specifically, we noted that research trends in this field changed drastically over the last 20 years, from the early development of ICIs, such as CTLA-4 inhibitors, to recent ones, such as PD-1 and PD-L1 blockers. Research focus has also gradually shifted from causes to side effects and adverse occurrences of treatments. Through translational research, clinical studies into the interactions of immune checkpoints with costimulatory molecules, cancer drive genes, and cancer hallmarks, as well as basic studies on the mechanism of action of ICIs have advanced from the early research stage with a focus on the mechanisms of immunotherapy, including the alteration of immune cells and immune molecules under ICI treatment. This suggests that this field of research is maturing. Further, clinical applications are being explored, including how to enhance efficacy, reduce adverse effects, and translate the findings to other specific cancer types. Currently, “plus chemotherapy” and “1^st^ line nivolumab” are new research hotspots with respect to the use of ICI for NSCLC treatment. Nivolumab restores the antitumor function of T cells. Further, nivolumab plus ipilimumab, as a combination immunotherapy, increases long-term survival in patients with advanced malignancies, including melanoma, renal cell carcinoma, malignant pleural mesothelioma, and NSCLC (NCT01844505, NCT02231749, NCT02899299) (Larkin et al., 2019; Motzer et al., 2020; Baas et al., 2021). First-line therapy with nivolumab and ipilimumab for advanced NSCLC drastically decreased progression in Part 1 of the randomized phase 3 CheckMate 227 trial compared to the platinum-doublet chemotherapy and significantly prolonged OS in patients with tumor PD-L1 expression ≥1% and disease-free survival in patients with a high tumor mutational load (NCT02477826, NCT02477826) (Hellmann et al., 2018; Hellmann et al., 2019). In recent years, it has been reported that in addition to PD1/PD-L1 and CTLA-4, a second group of co-inhibitory molecules, including lymphocyte-activation gene-3 (Lag-3 or CD223), T-cell immunoglobulin and mucin domain-3 (TIM-3), and T-cell immunoglobulin and ITIM domain (TIGIT), are also involved in controlling immunological responses, particularly at regions of tissue inflammation. Even though the expression patterns of LAG-3, TIM-3, and TIGIT largely overlap, their distinct signaling tails provide a foundation for both their distinct regulatory activities and the synergistic impact of medicines that target them in diseases (Goldberg and Drake, 2010; Sakuishi et al., 2010; Anderson Ana et al., 2016; Attanasio and Wherry, 2016). LAG-3 is highly similar to the CD4 protein in terms of structure and can bind MHC class II molecules (Andrews et al., 2017). Although there are active clinical trials of LAG-3-targeting therapeutics for NSCLC (NCT03625323, NCT01968109, NCT02750514, NCT02460224, and NCT03538028), it is still unknown how its expression is related to clinicopathologic factors and how it affects NSCLC prognosis. Additionally, TIM-3 is a type I transmembrane protein that includes a mucin stalk domain and a variable N-terminal Ig domain (Huang et al., 2015), and TIGIT is a receptor that modulates immunity and serves as an immunological checkpoint in both innate and adaptive immunity. In mice as well as in humans, TIGIT and PD-1 are co-expressed on tumor antigen-specific CD8 T cells and CD8 tumor-infiltrating lymphocytes (TILs) in cancer. Other ICIs also being investigated in clinical trials. For example, preliminary results have shown that sabatolimab plus spartalizumab therapy is well tolerated and shows signs of antitumor activity in NSCLC (NCT02608268) (Curigliano et al., 2021). Further, vibostolimab plus pembrolizumab shows anticancer effects in patients with advanced solid tumors, including advanced NSCLC, and is well tolerated (NCT02964013) (Niu et al., 2022). Molecular understanding of adaptive immunity over the past 10 years has made it possible to develop ICIs that are effective against a variety of malignancies; however, these often have unique side effects, known as immune-related adverse events (irAEs) (Naidoo et al., 2016; Sullivan and Weber, 2022). Considering the current research progress, we forecasted that in this developing field of study, expanding the indications for ICIs, exploring medication combinations, improving the efficacy of ICIs, reducing adverse effects, and screening biomarkers to predict efficacy and unfavorable effects will all likely become hot issues.

This study had some limitations. First, we used only one data source (WoSCC), indicating that the literature included may not be exhaustive; hence, some pertinent papers in other data sources may have been left out. Second, we only utilized papers written in the English language, which introduces some linguistic bias. Thus, the publications may not accurately represent all studies on ICIs for NSCLC. However, we believe our research will help readers gain a thorough understanding of this subject area.

## Abbreviations

AD, adenocarcinoma; CTLA-4, cytotoxic T-lymphocyte antigen 4; FDA, US Food and Drug Administration; ICI, immune checkpoint inhibitor; NSCLC, non-small cell lung cancer; OS, overall survival; PD-1, programmed cell death-1; PD-L1, PD ligand 1; PFS, progression-free survival; SCLC, small cell carcinoma; WoSCC, Web of Science Core Collection.
